# Melatonin downregulates the increased hepatic alpha-fetoprotein expression and restores pancreatic beta cells in a streptozotocin-induced diabetic rat model: a clinical, biochemical, immunohistochemical, and descriptive histopathological study

**DOI:** 10.3389/fvets.2023.1214533

**Published:** 2023-08-16

**Authors:** Khalaf F. Alsharif, Asmaa A. Hamad, Mohamed A. Alblihd, Fatma Abo Zakaib Ali, Sherine Ahmed Mohammed, Abdulrahman Theyab, Osama M. Al-Amer, Malik Saad Almuqati, Abdulraheem Ali Almalki, Alaa Jameel A. Albarakati, Khalid J. Alzahrani, Ashraf Albrakati, Mohammad Hamed Albarakati, Doaa Abass, Maha S. Lokman, Ehab Kotb Elmahallawy

**Affiliations:** ^1^Department of Clinical Laboratories Sciences, College of Applied Medical Sciences, Taif University, Taif, Saudi Arabia; ^2^High Altitude Research Center, Taif University, Taif, Saudi Arabia; ^3^Department of Biology, College of Science, Taif University, Taif, Saudi Arabia; ^4^Department of Medical Microbiology and Immunology, College of Medicine, Taif University, Taif, Saudi Arabia; ^5^Department of Pathology and Clinical Pathology, Faculty of Veterinary Medicine, Sohag University, Sohag, Egypt; ^6^Department of Histology, Faculty of Medicine, Sohag University, Sohag, Egypt; ^7^Department of Laboratory and Blood Bank, Security Forces Hospital, Mecca, Saudi Arabia; ^8^College of Medicine, Al-Faisal University, Riyadh, Saudi Arabia; ^9^Department of Medical Laboratory Technology, Faculty of Applied Medical Sciences, University of Tabuk, Tabuk, Saudi Arabia; ^10^Genome and Biotechnology Unit, Faculty of Sciences, University of Tabuk, Tabuk, Saudi Arabia; ^11^Department of Laboratory, King Fahad Armed Forces Hospital, Jeddah, Saudi Arabia; ^12^Department of Clinical Laboratory Sciences, College of Applied Medical Sciences, Taif University, Taif, Saudi Arabia; ^13^Surgery Department, College of Medicine, Al-Qunfudah Branch, Umm Al-Qura University, Mecca, Saudi Arabia; ^14^Department of Human Anatomy, College of Medicine, Taif University, Taif, Saudi Arabia; ^15^Cardiology Department, King Abdullah Medical Complex, Jeddah, Saudi Arabia; ^16^Zoology Department, Faculty of Sciences, Sohag University, Sohag, Egypt; ^17^Department of Biology, College of Science and Humanities in Al-Kharj, Prince Sattam Bin Abdulaziz University, Al-Kharj, Saudi Arabia; ^18^Department of Zoology and Entomology, Faculty of Science, Helwan University, Cairo, Egypt; ^19^Departamento de Sanidad Animal, Grupo de Investigación en Sanidad Animal y Zoonosis (GISAZ), Facultad de Veterinaria, Universidad de Córdoba, Córdoba, Spain; ^20^Department of Zoonoses, Faculty of Veterinary Medicine, Sohag University, Sohag, Egypt

**Keywords:** melatonin, diabetes, STZ, histopathology, liver, alpha-fetoprotein expression

## Abstract

**Background:**

Diabetes mellitus (DM) is a chronic metabolic disorder. Hepatopathy is one of the serious effects of DM Melatonin (MT) is a potent endogenous antioxidant that can control insulin output. However, little information is available about the potential association between melatonin and hepatic alpha-fetoprotein expression in diabetes.

**Objective:**

This study was conducted to assess the influence of MT on diabetes-related hepatic injuries and to determine how β-cells of the pancreas in diabetic rats respond to MT administration.

**Materials and methods:**

Forty rats were assigned to four groups at random (ten animals per group). Group I served as a normal control group. Group II was induced with DM, and a single dose of freshly prepared streptozotocin (45 mg/kg body weight) was intraperitoneally injected. In Group III, rats received 10 mg/kg/day of intraperitoneal melatonin (IP MT) intraperitoneally over a period of 4 weeks. In Group IV (DM + MT), following the induction of diabetes, rats received MT (the same as in Group III). Fasting blood sugar, glycosylated hemoglobin (HbA1c), and serum insulin levels were assessed at the end of the experimental period. Serum liver function tests were performed. The pancreas and liver were examined histopathologically and immunohistochemically for insulin and alpha-fetoprotein (AFP) antibodies, respectively.

**Results:**

MT was found to significantly modulate the raised blood glucose, HbA1c, and insulin levels induced by diabetes, as well as the decreased alanine aminotransferase (ALT) and aspartate aminotransferase (AST). Furthermore, MT attenuated diabetic degenerative changes in the pancreas and the hepatic histological structure, increased the β-cell percentage area, and decreased AFP expression in the liver tissue. It attenuated diabetes-induced hepatic injury by restoring pancreatic β-cells; its antioxidant effect also reduced hepatocyte injury.

**Conclusion:**

Collectively, the present study confirmed the potential benefits of MT in downregulating the increased hepatic alpha-fetoprotein expression and in restoring pancreatic β-cells in a streptozotocin-induced diabetic rat model, suggesting its promising role in the treatment of diabetes.

## 1. Introduction

Diabetes mellitus (DM) is a chronic metabolic disease. It affects millions of individuals worldwide. In 2030, 366 million people are predicted to have DM worldwide (1). Many experimental models have become available for studying the evolution of and the complications associated with types 1 and 2 DM (2). The streptozotocin (STZ)-induced experimental model of diabetes has been widely used. Streptozotocin passes into pancreatic cells via glucose transporter 2 channels in the cell membrane, where it causes β-cell damage, resulting in decreased insulin levels, and hyperglycemia in experimental animals (1). Oxidative stress is considered an important contributor to complications associated with DM. During oxidative stress, reactive oxygen species (ROS) damage cellular proteins. Multiple severe and sometimes fatal consequences, including neuropathy, nephropathy, retinopathy, vasculopathy, and hepatopathy, can arise from the association between DM and substantial abnormalities in lipid and protein metabolisms (3). Liver injury in patients with diabetes commonly occurs due to oxidative stress induced by hyperglycemia. Alpha-fetoprotein (AFP) is a single-stranded glycoprotein primarily produced by the fetal liver and yolk sac. The level of AFP declines rapidly after birth and remains low throughout life. It is reactivated during liver regeneration and hepatocarcinogenesis (4). Adult hepatocytes can re-express AFP when they act as functional stem cells and can regenerate upon exposure to injury (5).

Melatonin (MT), also known as 5-methoxy-N-acetyltryptamine, is an endogenous antioxidant produced and secreted by the pineal gland. MT levels decline with increasing age. This hormone regulates various physiological and pathological conditions in a daily rhythm (6–13). Plasma concentrations of MT were shown to be significantly lower in diabetic rats and people with diabetes compared to healthy rats and humans. Furthermore, MT performs actions that are anti-apoptotic and anti-inflammatory (7). Through its impact on insulin secretion regulation both *in vivo and in vitro*, MT can impact metabolic diseases such as diabetes (13–15). Specifically, MT regulates insulin release via G-protein-coupled melatonin receptors present on β-cells, in particular MT1 and MT2 receptors (16). Hepatic complications of DM provide a challenge for medical practitioners (17). Upon reviewing the available literature, little information was found about the potential effect of MT on the regulation of hepatic alpha-fetoprotein expression and pancreatic beta cells in diabetes.

This study was conducted to assess the effect of MT on glucose metabolism and liver enzymes in diabetic rats and to confirm these effects histopathologically and immunohistochemically by measuring the area of β-cells of the pancreas and assessing diabetes-induced hepatocellular injury, as indicated by the AFP expression.

## 2. Material and methods

### 2.1. Ethical considerations

This study and all experimental methods were carried out in accordance with the Declaration of Helsinki and the ethics committee's rules of Taif University in Taif, Saudi Arabia (approval number HAO-02-T-105).

### 2.2. Materials

#### 2.2.1. Chemicals and drugs

Streptozotocin powder and trisodium citrate dihydrate were purchased from Sigma-Aldrich (St. Louis, MO, USA). N-acetyl-5-methoxytryptamine (melatonin) was purchased from FAGRON (Cat# 420 33457-24, Fagron, Nazareth, Belgium). Furthermore, AFP polyclonal antibody was purchased from Thermo Fisher Scientific, Neomarks, Fremont, USA, PA5-21004. Dilution (1:100). Monoclonal anti-insulin antibody was purchased from Invitrogen [INS05 (2D11-H5)], Catalog # MA5-12037. Dilution: (1:100).

### 2.3. Animals

Forty mature male Wister albino rats aged 7 weeks and weighing 200 ± 25 g were used in the study. Five rats per cage were housed in specialized, clean, and pathogen-free stainless-steel cages, which also had a 50:50 humidity ratio and a 12-h light/dark cycle. The temperature was kept at 23 ± 2°C. Rats were fed a standard pellet diet and water *ad libitum* throughout the experimental period. To maintain a clean environment, the bedding was replaced often. They had a week to acclimatize before the experiment was started.

### 2.4. Experimental approach

Rats were kept under strict observation during the acclimatization week. Following adaptation, they were divided into four separate, isolated groups, each consisting of ten animals. Group I: Normal control (*n* = 10): These rats were fed regular rat food and given access to water. Animals in Group II were made to fast overnight (DM group; *n* = 10) (12 h before diabetes induction).

### 2.5. Induction of diabetes

A single intraperitoneal injection of freshly prepared streptozotocin (45 mg/kg body weight) in 0.1 M cold citrate buffer (pH 4.5) was used to induce diabetes (18, 19). The rats were allowed free access to food and water after the injection, and a 15% glucose solution was provided to drink overnight to prevent hypoglycemia. Within 3–6 days of STZ treatment, hyperglycemia was observed, as recorded in previous studies (20–23). Consequently, the blood sugar levels were checked 3–6 days after STZ treatment. Polydipsia, polyuria, and blood glucose levels recorded 72 h after STZ injection in a sample of blood drawn via a tail prick using a glucometer (On Call Plus, ACON Laboratories, Germany) were used to diagnose diabetes. Only STZ-injected rats with blood glucose levels of 250 mg/dl or more were considered diabetic and were selected for the study.

**Group III: Melatonin group (MT)** (*n* = 10): Melatonin was dissolved in DMSO (1% w/v) before injecting IP into rats at a dose of 10 mg/kg/day for 4 weeks (18, 21).

**Group IV: Diabetes (DM)** **+** **melatonin group (MT)** (*n* = 10): Rats were administered IP MT (identical to MT group III) after STZ treatment (similar to diabetic control positive group II). Twelve hours after the final administration of MT, all rats were euthanized, and their blood and tissue samples were collected.

### 2.6. Methods

#### 2.6.1. Animal body weight measurements

Body weight was recorded weekly, and the final body weights of all experimental rats were recorded at the time of euthanasia.

#### 2.6.2. Liver weight measurement

All experimental rats were euthanized at the completion of the study, and the liver weights were recorded.

#### 2.6.3. Sample collection

##### 2.6.3.1. Blood samples used for determining the fasting blood sugar level

The animals' tails were sterilized with 10% alcohol, and blood samples were obtained by tail pricking and letting the blood touch the test strip, which was inserted into a calibrated glucose meter (On Call Plus Glucometer, ACON Laboratories, Germany). The method employed was modified from those of other studies described elsewhere (23, 24). A direct reading in mg/dL was obtained upon reading after 5 s.

#### 2.6.4. Whole blood samples

At the termination of the experimental period, rats were euthanized, and individual blood samples from each group were taken in EDTA-containing, dry, clean tubes for assessing glycosylated hemoglobin (HbA1c) (25).

#### 2.6.5. Biochemical analysis

##### 2.6.5.1. Fasting blood glucose level assessment

As previously mentioned in the experimental design, the fasting blood glucose level was assessed continuously during the experiment (24).

##### 2.6.5.2. Assessment of serum biochemical parameters

Cumulative blood sugar levels were determined using the Arkray Automatic Glycohemoglobin Analyzer ADAMS A1c HA-8190V, ADAMS A1c, HA-8190V, which is a fully automated glycohemoglobin analyzer. The HbA1c analyzer is based on high-performance liquid chromatography (HPLC). HA-8190V automatically detects and separates variant hemoglobins (25). Insulin concentration in serum was determined using a rat insulin enzyme immunoassay (Société de Pharmacologie et d'Immunologie - BIO, France), according to the manufacturer's instructions (26). Serum biochemical parameters including ALT and AST were assessed by employing the enzymatic approach for the quantitative assessment of blood glucose (BG) (the glucose oxidase method) (27). The kinetic technique was employed to determine both ALT and AST (27, 28). Spectrophotometric methods (5010 V5+, semi-automatic photometer, RIELE, Germany) were employed to analyze the concentrations of all the aforementioned serum biochemical variables in accordance with the manufacturer's instructions.

##### 2.6.5.3. Histopathological examination

At the conclusion of the experiment, the animals were euthanized, and tissue samples from the pancreas and liver were obtained, dissected, and promptly fixed in 10% formalin for 24 h. They were then dehydrated in graded alcohol series, cleared in xylene, and embedded in paraffin using the standard technique (29). Tissue sections were cut at a thickness of 3 μm. Hematoxylin and eosin (H&E) stains were used (29) for general histopathological examination. All sections were examined and captured using an OLYMPUS SC52 camera that was adapted for use with the light microscope OLYMPUS CX43.

##### 2.6.5.4. Immunohistochemical studies

Insulin antibodies for β-cells in islets of Langerhans and the AFP antibody for hepatocyte regeneration were stained and examined. Tissue slides were deparaffinized in xylene and then rehydrated in descending grades of alcohol. A labeled streptavidin-biotin method was employed, and the anti-polyvalent HRD/DAP plus labvision detection system was used. Slides were boiled in 10 mmol/l citrate buffer (pH 6.2) for two cycles of 3 min each in a microwave oven for antigen retrieval. The lost buffer was replaced in between cycles. The endogenous peroxidase was blocked with 2% hydrogen peroxide for 5 min. Liver sections were incubated with the primary AFP antibody. The polyclonal antibody was purchased from Thermo Scientific, Neomarks, Fremont, USA, PA5-21004 Dilution (1:100). Pancreas sections were incubated with the primary anti-insulin antibody {monoclonal was purchased from Invitrogen, USA [INS05 (2D11-H5)], Catalog # MA5-12037 Dilution: 1:100}.

After incubation of sections with the primary antibody overnight at 4°C, the sections were washed in phosphate-buffered saline (pH 7.2) and incubated first with biotinylated secondary antibodies and then with the avidin–biotin complex. Incubations were performed at room temperature, and the staining was visualized with diaminobenzidine and chromogen 1:25. Then, the sections were counterstained with Mayer's hematoxylin solution. They were cleared in xylene and mounted with a cover slip. The negative control slides were prepared by omitting primary antibodies and using non-immunized goat serum. Positive cells showed brown cytoplasmic staining. Positive control: The insulin was used to stain β-cells in islets of Langerhans, and the AFP antibody was used to stain hepatocytes in liver cancer (30).

##### 2.6.5.5. Histomorphometric assessments

Organ histology analysis was performed by assigning a score based on the severity of damage observed in each category of the tissue under examination: 0 = no lesions; 1 = mild (1 to 25%); 2 = moderate, (26 to 45%); and 3 = severe (>45%), as previously mentioned (31, 32). Using a light microscope (Leica ICC50 Wetzlar, Germany) at the Histology Department, Faculty of Medicine, ten non-overlapping high-power fields (×400) for each section in all animals in each group were taken and analyzed using Image J 1.51n software [National institutes of health USA Java 1.8.0_66 (32-bit)]. This was performed to detect the β-cell area percentage (the percentage of the insulin-positive area over the islet area) was calculated by dividing the area of all insulin-positive cells by the islet area and by multiplying it by 100. In three consecutive serial sections of the pancreas (200 um apart) (33), the percentages of the AFP expression area were calculated in each examined field.

##### 2.6.5.6. Statistical analysis

The measurements obtained from the experimental groups were statistically estimated using GraphPad Prism, version 5 (San Diego, California, USA) using one-way ANOVA with Tukey's *post hoc* multiple-comparison tests to define the statistical significance between the groups. The data were expressed as mean ± standard deviation (SD), and a *p*-value of *P* < 0.05 was considered significant (34, 35).

## 3. Results

### 3.1. Diabetic induction, body weight, and liver weight measurements

After diabetic induction by STZ, type 1 DM was recorded in the animals within 72 h. Furthermore, typical DM symptoms, including polyphagia, polydipsia, polyuria, and unexplained significant (*P* ≤ 0.05) weight loss, were recorded in the diabetic untreated rats. The animals' body weight started to reduce from the end of the first week after diabetic induction until the end of the experimental period before the scarification time (Table 1). The decrease in body weight was much more significant (*P* ≤ 0.05) in the DM untreated group than in the DM+MT treated group compared with the control group (Table 1).

**Table 1:** Comparison of the body weight between experimental groups in the DM model.

**Groups**	**Body weight (g) (Mean** ±**SD)**				
	**Initial body weight (before the experiment)**	**After 1**^st^ **week**	**After 2**^nd^ **week**	**After 3**^rd^ **week**	**Final body weight (Before euthanasia)**
Control	177.1 ± 15.41	197.4 ± 13.45	196.5 ± 16.48	196.5 ± 16.48	207.6 ±19.50
Diabetic (DM)	192.4 ± 12.53	157.3 ± 19.51^**^	125.9 ± 20.10^***^	146.7 ± 12.55^**^	144.4 ± 13.48^***^
MT	166.5 ± 6.028	199.6 ± 23.08^ns^	220.6 ± 9.072^ns^	204.8 ± 12.13^ns^	204.6 ± 10.11^ns###^
DM+MT	190.3 ± 24.12	174.2 ± 26.72^*^	168.0 ± 28.06^*^	177.2 ± 31.19^ns^	176.4 ± 24.46^*#^

Values are expressed in Means ± SD. Significant differences (control vs. DM and MT control vs. DM+MT are marked by different asterisks), (DM vs. MT control and DM+MT groups are marked by #) through one-way ANOVA with Tukey's *post-hoc* test: ns; non-significant, ^*#^*P* ≤ 0.05, ^**^P ≤ 0.01, and ^***^^###^*P* ≤ 0.001).

A comparison of liver weight measurements showed non-significant (*P* ≤ 0.05) changes between the experimental groups (Table 2). However, the mean value of liver weights relative to the body weight of experimental rats at the scarification time revealed an increase in liver weights in the DM untreated group, which was much more in this group than in the DM+MT treated group, all compared with the control group (Tables 1, 2).

**Table 2:** Comparison of the liver weight between experimental groups in the DM model.

**Groups**	**Liver weight (g)**
Control	5.923 ± 0.6472
Diabetic (DM)	5.498 ± 0.6132^ns^
MT	5.210 ± 0.3713^ns^
DM+MT	5.182 ± 1.178^ns^

Values are expressed in Means ± SD. Significant differences vs. the control group (*P* ≤ 0.05) through one-way ANOVA with Tukey's *post-hoc* test: ns, non-significant.

### 3.2. Results of serum biochemical assessments

Statistical analysis of fasting blood sugar during the first, second, and third weeks of the experiment showed that blood sugar levels in both the DM group and the DM group treated with MT were significantly (*P* ≤ 0.05) increased when compared with the control negative and MT groups (Table 3). At the end of the fourth week and before scarification, the mean value of the DM + MT treated group was significantly (*P* ≤ 0.05) decreased when compared with the DM untreated group, and it started to approach the blood sugar levels of the control and MT control groups. However, the increase in blood sugar levels was still significant when compared with the two control groups (Table 3).

**Table 3:** Comparison of the fasting blood glucose level between experimental groups in the DM model.

**Groups**	**Fasted blood glucose level (mg/dl) after diabetic induction (Mean** ±**SD)**			
	**1^st^ week**	**2^nd^ week**	**3^rd^ week**	**4^th^ week (Before euthanasia)**
Control	86.00 ± 5.888	64.33 ± 5.132	51.40 ± 3.647	66.20 ± 7.190
Diabetic (DM)	420.3 ± 52.67^***^	381.2 ± 27.34^***^	483.3 ± 44.91^***^	362.0 ± 123.1^***^
MT	105.0 ± 5.196^ns^	97.75 ± 5.315^ns^	76.00 ± 34.60^ns^	66.25 ± 3.775^ns###^
DM+MT	395.3 ± 36.47^***^	365.0 ± 45.49^***^	331.5 ± 28.67^***^	183.3± 40.95^*###^

Values are expressed in Means ± SD. Significant differences (control vs. DM, MT control and DM+MT groups are marked by different asterisks), (DM vs. MT control and DM+MT groups are marked by #) through one-way ANOVA with Tukey's *post-hoc* test: ns; non-significant, ^*^P ≤ 0.05, ^***^^###^*P* ≤ 0.001.

The DM untreated group had the most significant HbA1c level, which was considerably (*P* ≤ 0.05) higher than the control negative and MT control groups. This level also reflected the diabetic condition of the rats in this group. The mean value of the cumulative blood sugar level in the DM+MT treated group was significantly (*P* ≤ 0.05) higher when compared with the control and MT control groups and significantly (*P* ≤ 0.05) lower when compared with the DM group (Table 4).

**Table 4:** Comparison of the serum biochemical profile of the cumulative blood sugar value (HbA1C), insulin, ALT, and AST between experimental groups in the DM model.

**Groups**	**Cumulative blood sugar (HbA1C) value (Mean ±SD)**	**Insulin (uIU/ml)**	**ALT (U/L)**	**AST (U/L)**
Control	74.32± 5.584	1.398 ± 0.2179	56.24 ± 2.309	157.3 ± 2.275
Diabetic (DM)	212.4 ± 12.06^***^	0.5700 ± 0.09772^***^	140.4 ± 4.248^***^	273.7 ± 47.40^***^
MT	72.68 ± 8.266^ns###^	1.352 ± 0.1999^ns^	49.96 ± 5.489^ns^	169.3 ± 2.296^ns^
DM+MT	123.3 ± 25.67^**###$$^	0.9460 ± 0.1106^**^	92.22 ± 4.462^***###^	191.0 ± 27.51^ns###^

Values are expressed in Means ± SD. Significant differences (control vs. DM, MT control, and DM+MT groups are marked by different asterisks), (DM vs. MT control and DM+MT groups are marked by #), (MT control vs. DM+MT groups are marked by $) through one-way ANOVA with Tukey's *post-hoc* test: ns; non-significant, ^*^P ≤ 0.05, ^**^^,^^$$^*P* ≤ 0.01, ^***^^###^*P* ≤ 0.001.

In this study, the serum concentration of insulin was significantly decreased in rats with DM when compared with control rats (Table 4), but no significant change was recorded in this variable between the MT and control groups. Additionally, the DM+MT group had significantly (*P* ≤ 0.05) more insulin than the DM group, which was another noteworthy difference (Table 4).

Alanine aminotransferase was significantly elevated in the DM group compared with the control group. No significant change in ALT was recorded between the MT and control groups. However, the DM+MT group showed a significant decrease in ALT compared with the DM group (Table 4). Concentrations of AST demonstrated a significant (*P* ≤ 0.05) increase in the DM and MT groups compared with the control group (Table 4). In contrast, AST concentrations demonstrated a significant (*P* ≤ 0.05) decrease in the DM+MT group compared with the DM group (Table 4).

### 3.3. Histopathological assessment

#### 3.3.1. Pancreatic tissue

**Figure 1:**
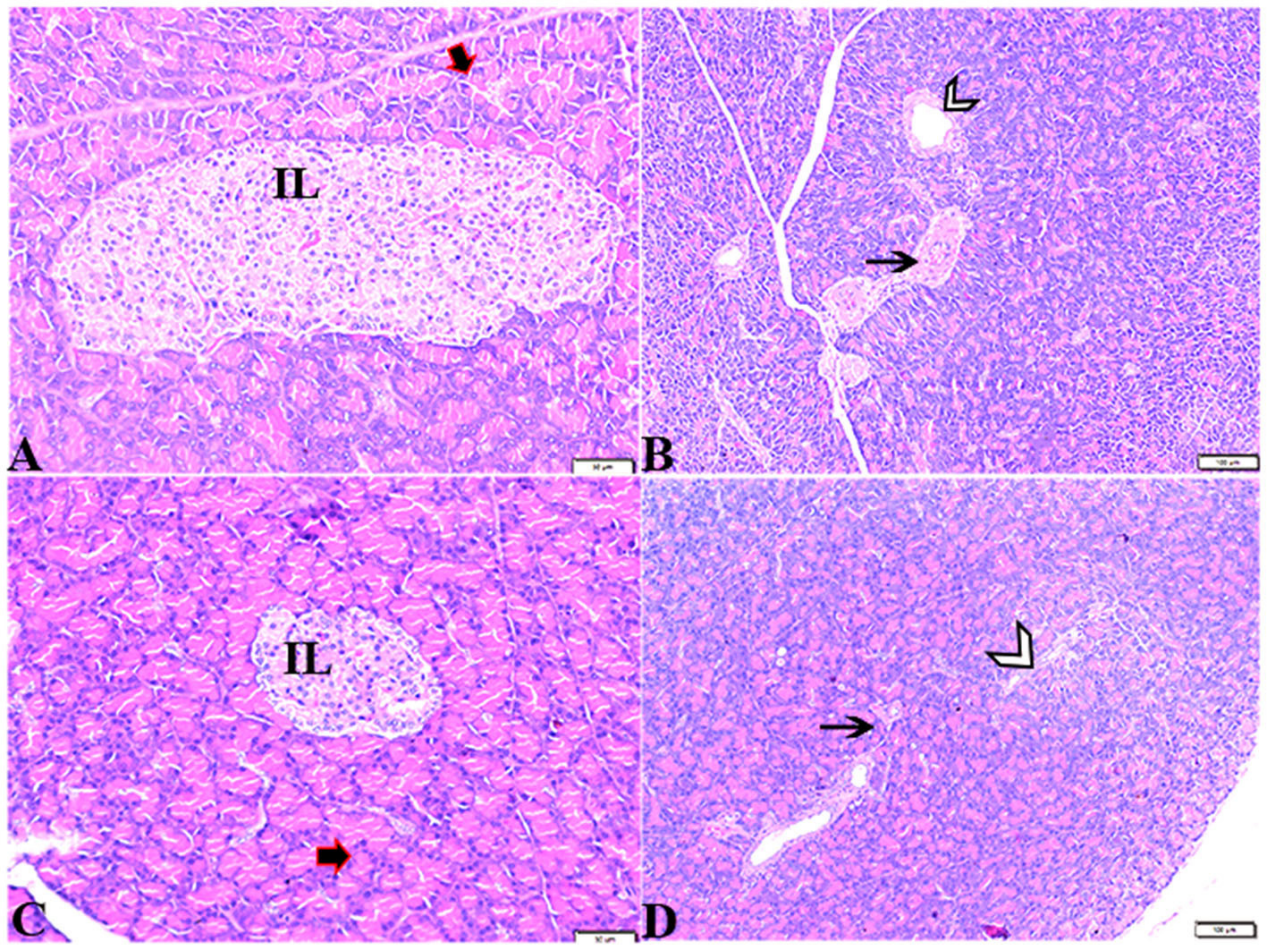
Photomicrograph of rat pancreas sections stained with H&E stain from the control negative **(A, B)** and MT control groups **(C, D)** demonstrating the following: normal pancreatic structure and architecture in the form of **(A, C)** normal-sized islets of Langerhans showing the normal density of islet cells (IL) and normal exocrine acinar cell (thick arrows). **(B, D)** Normal intralobular duct (arrowheads) and normal blood vessels (thin arrows).

**Figure 2:**
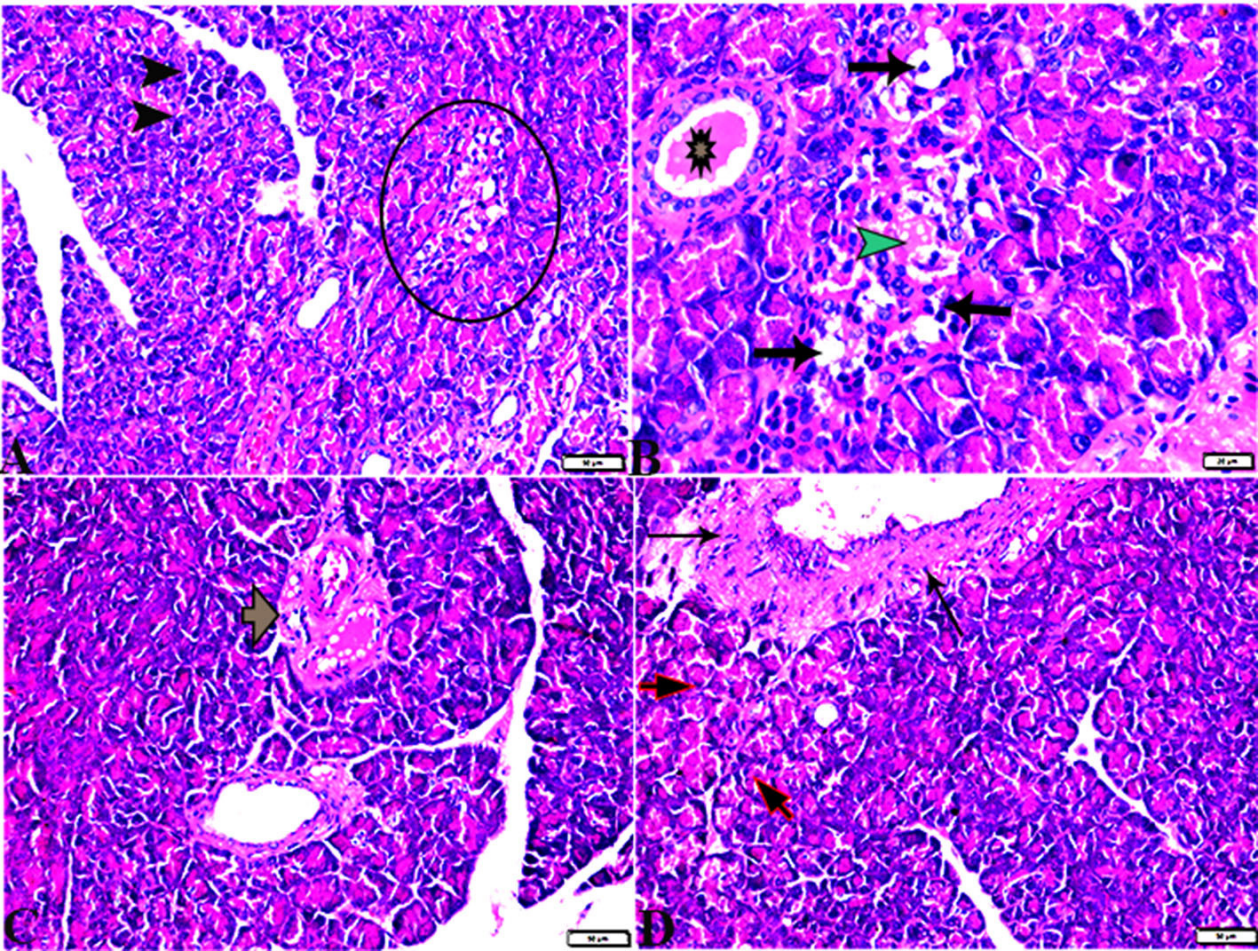
Photomicrographs of pancreas sections stained with H&E stain **(A–D)** from the DM group showing the following: **(A)** Islets of Langerhans, which undergo depletion and necrobiotic changes in their constituent cells (Circle), and necrotic acinar cells (arrowheads). **(B)** Islets of Langerhans showing vacuolated degeneration in most cells (arrows), capillary hemorrhage inside islets green arrowhead), and the intralobular duct dilated with secretion (Star). **(C)** Dilated and congested blood vessels with a thick vascular wall (arrow). **(D)** Thickening of the wall of the blood vessel (thin arrows), the exocrine acinar cells showing degeneration and dissociation (red arrows).

**Figure 3:**
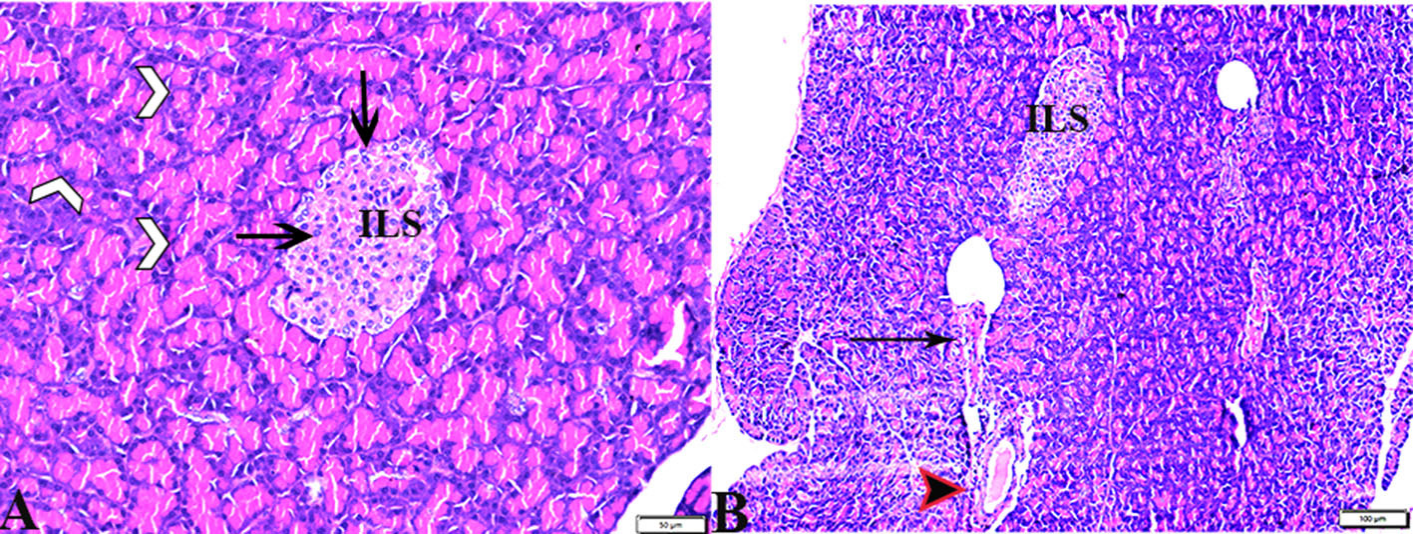
Photomicrograph of pancreas sections stained with H&E stain from the DM+MT treated group showing the following: **(A)** Well-defined islets of Langerhans with proliferated cell population (arrow, ILS) in between normal pyramidal acidophilic pancreatic acini (white arrowheads). **(B)** Normal vascular structure (arrowhead) and normal intralobular duct (arrows).

The pancreatic endocrine and exocrine parenchymal structures were revealed by microscopic examination of pancreatic tissue sections from the experimental groups in both control negative and MT treated rats. The endocrine islets of Langerhans were observed to be substantial pale oval regions with a distinct border between them. They consisted of numerous small, pale β-cells and a few large, acidophilic, and rounded α-cells, which were arranged around tiny blood capillaries in between a large number of pyramidal acinar cells (Figures 1A–D). Several pathological findings were obtained from the sections of the DM rats, including distorted pancreatic lobules and necrobiotic changes in the islets of Langerhans cells in which the nucleus was pyknotic and other places were lysed and disappeared, and the destroyed cells were replaced by fat tissue. Additionally, capillary congestion in between islet cells was also observed (Figures 2A, B). The exocrine parenchymal portion in this group showed atrophy, degeneration, and dissociation of some exocrine acini (Figures 2A, D). The vascular system of the pancreatic tissue from this group generally revealed severe congestion with a very thick wall (Figures 2C, D). Multiple normal pancreatic lobules with thinly spaced interlobular septa were observed in the DM+MT rats, which was improved through concurrent MT administration to DM rats in Group IV. The exocrine parenchyma had multiple pancreatic acini with typical pyramidal acinar cells, and the islets of Langerhans had normal cellular density and cells with enhanced morphological appearance (Figure 3A). Moreover, the blood vessels had a normal structure (Figure 3B).

#### 3.3.2. Quantitative and semi-quantitative histomorphometry studies

##### 3.3.2.1. Pancreatic tissue scoring

**Figure 4:**
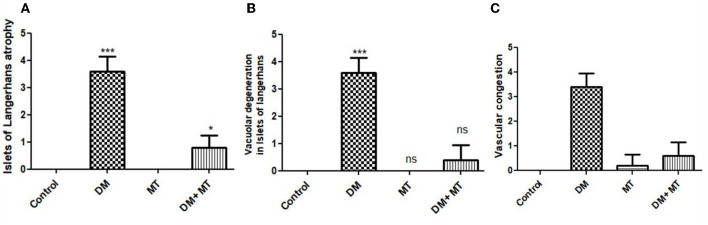
Histomorphometry graph showing semiquantitative measurements of pancreatic tissue sections among the experimental groups: **(A)** Islets of Langerhans atrophy, **(B)** Vacuolar degeneration in the islets of Langerhans, and **(C)** Vascular congestion. Data are expressed as means ± standard deviations. Significant differences vs. the control group are marked by different asterisks through one-way ANOVA with Tukey's *post-hoc* test: *P ≤ 0.05, ***P ≤ 0.001.

Comparing the DM group with other groups, pancreatic histomorphometric results showed significant (*P* ≤ 0.05) cell damage in the pancreatic tissue, which was characterized by atrophy in the islets of Langerhans, vacuolar degeneration in the islet cells, and vascular congestion (Figures 4A–C). However, compared with the control groups, MT-treated diabetic rats displayed a non-significant (*P* ≤ 0.05) difference.

#### 3.3.3. Liver

**Figure 5:**
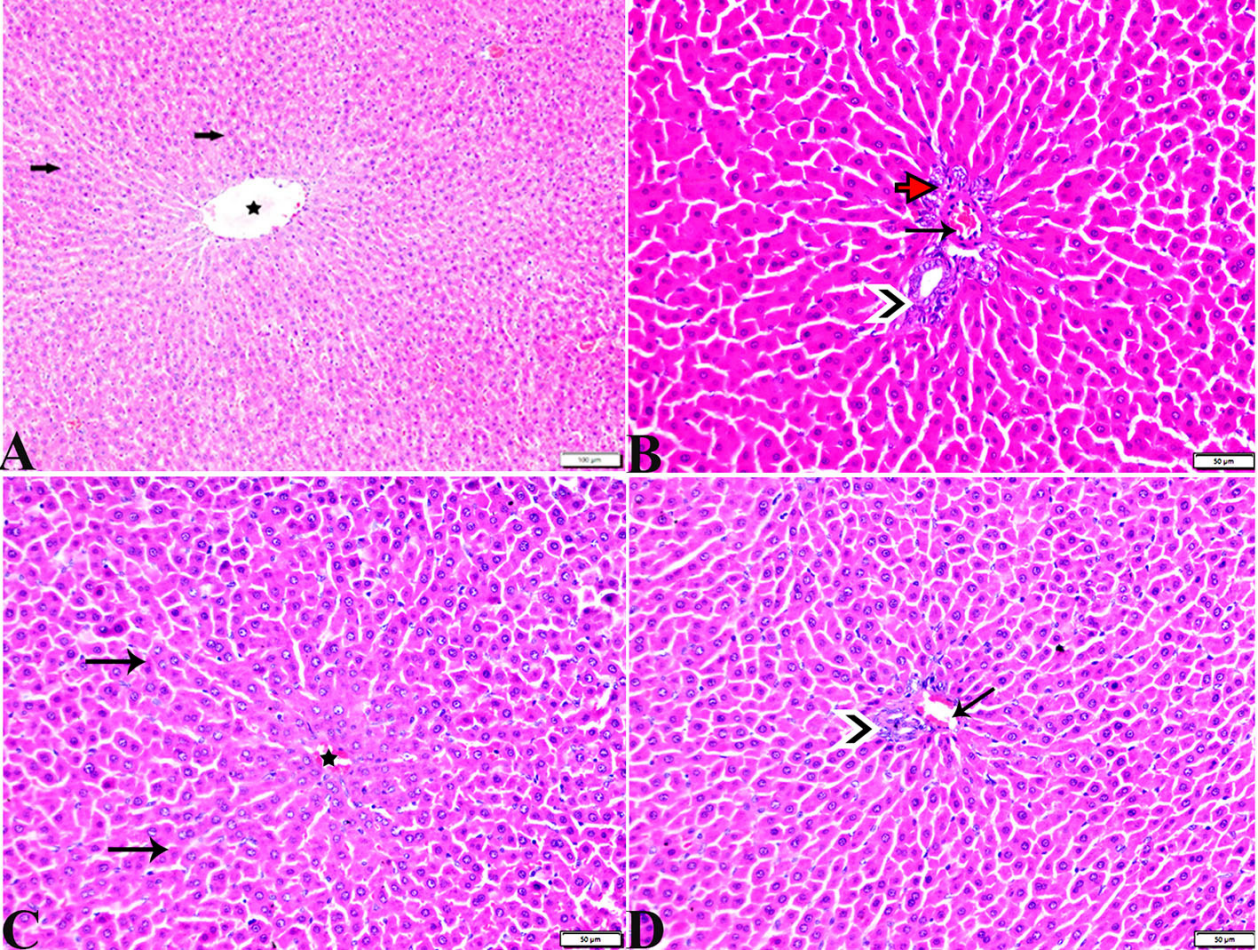
Photomicrographs of hepatic tissue sections stained with H&E stain from the control negative **(A, B)** and MT control groups **(C, D)**, showing the following: normal histological hepatic architectures compromising in **(A, C)**. Normal central veins (stars), normal hepatocytes arrangement, and a structure with normal vesicular nucleus (arrows). **(B, D)** Normal portal triad structure present in the normal portal vein (arrows), hepatic artery (red arrow), and bile duct (arrowheads).

**Figure 6:**
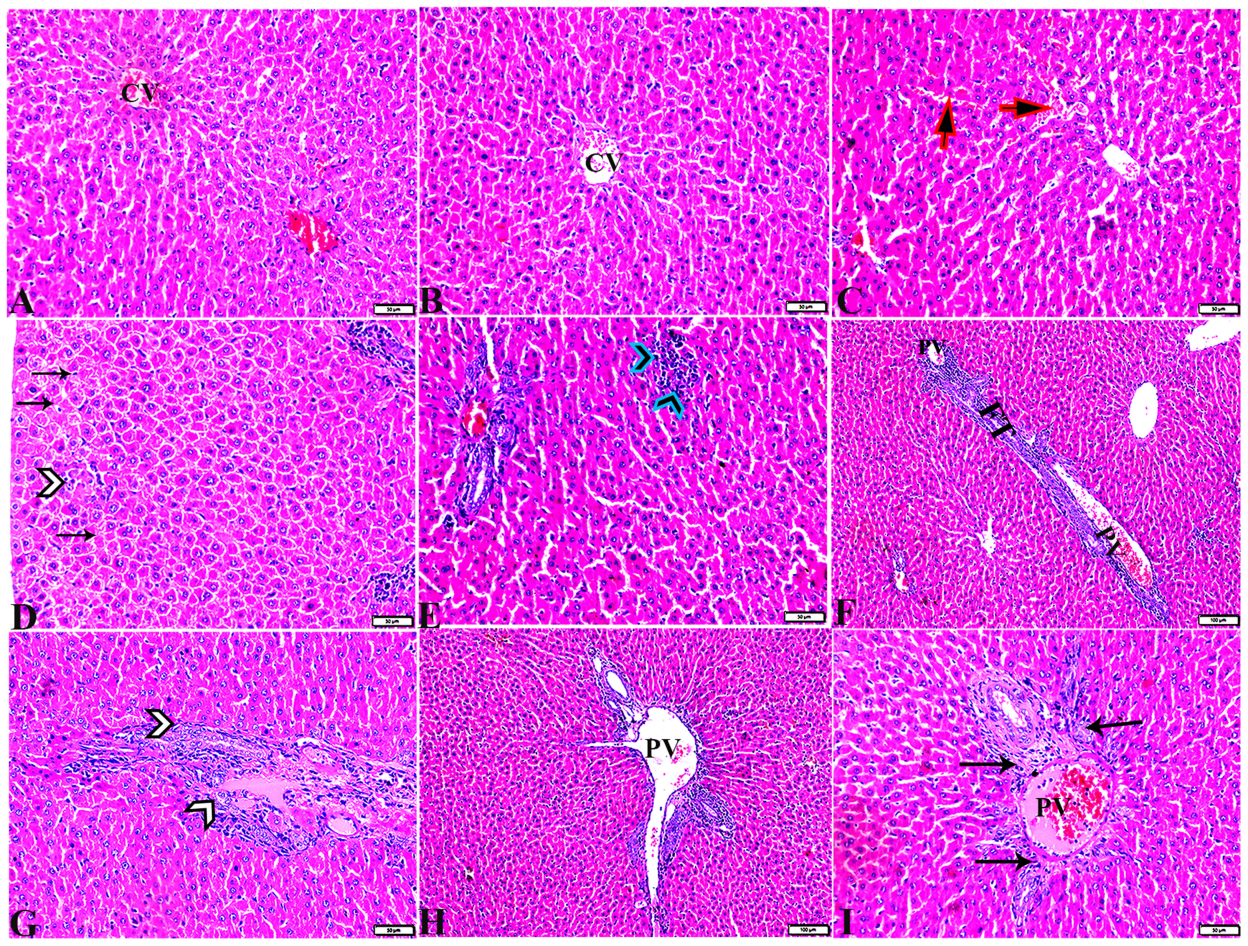
Photomicrographs of liver sections stained with H&E stain from the DM untreated group showing the following: **(A, B)** Congested central veins (C.V). **(C)** Congestion and dilatation in hepatic sinusoids (arrows). **(D)** Hepatocellular degeneration (arrows) and mononuclear inflammatory cellular infiltration (arrowhead). **(E)** Focal mononuclear cellular aggregation (arrowheads). **(F)** Fibrous tract (FT) connecting between portal areas and surrounding the congested portal veins (PV). **(G)** Inflammatory cellular infiltration in the fibrous tract (arrowheads). **(H, I)** Portal areas showing the following: Severe dilatation and congestion in portal veins (PV) and periportal fibrosis infiltrated with inflammatory cells (arrows).

**Figure 7:**
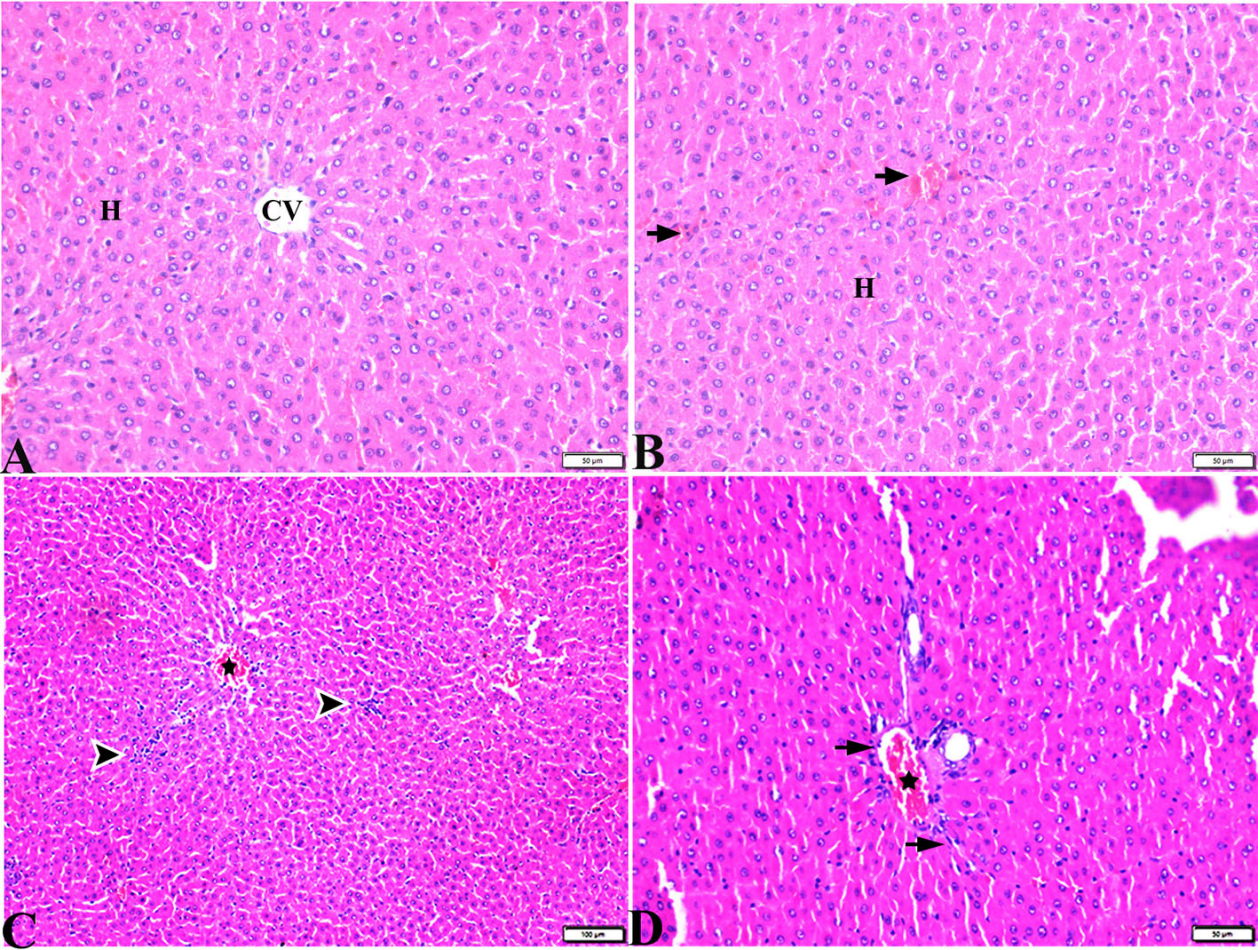
Photomicrographs of liver sections stained with H&E stain from DM+MT treated rats showing the following: **(A, B)** Normal central vein (CV), normal hepatocellular architecture (H), and mild interstitial hemorrhage (arrows). **(C)** Normal central vein (star) and mild mononuclear cellular aggregation (arrowheads). **(D)** Portal area; mild congested portal vein (star) surrounded with mild fibrosis (arrows).

Pathological changes in the liver tissue were observed in the experimental groups but not in the control groups, which exhibited an intact hepatic architecture in hepatic lobules, normal central veins, normal hepatocytes arrangement, and a structure with a normal vesicular nucleus. A normal portal triad structure was present in the normal portal vein, hepatic artery, and bile duct (Figures 5A–D). Histological changes were not observed in the hepatic tissue of rats from the control negative group when compared with the MT control group throughout the experimental period (Figures 5C, D). Livers of DM untreated rats demonstrated central veins, and hepatic sinusoids between hepatic cords were dilated and congested (Figures 6A–C). Furthermore, hepatocytes showed degeneration with diffuse mononuclear inflammatory cellular infiltration in some areas (Figure 6D), and focal mononuclear cellular aggregation was also observed (Figure 6E). The interlobular vein was distended and congested with blood. Furthermore, periportal fibrosis was observed (Figures 6F–I). The fibrous tract extended as a bridge connecting portal areas and surrounding the congested portal veins with inflammatory cellular infiltration (Figures 6F–I). Additionally, liver tissue sections from DM rats treated with MT demonstrated marked improvement in hepatic architectures present in normal central veins and hepatocytes (Figure 7A). Mild interstitial congestion and mild mononuclear cellular aggregation were observed (Figures 7B, C). The portal area demonstrated a marked improvement in its structure, except for a mild congested portal vein surrounded with mild fibrosis (Figure 7D).

#### 3.3.4. Hepatic tissue damage scoring

**Figure 8:**
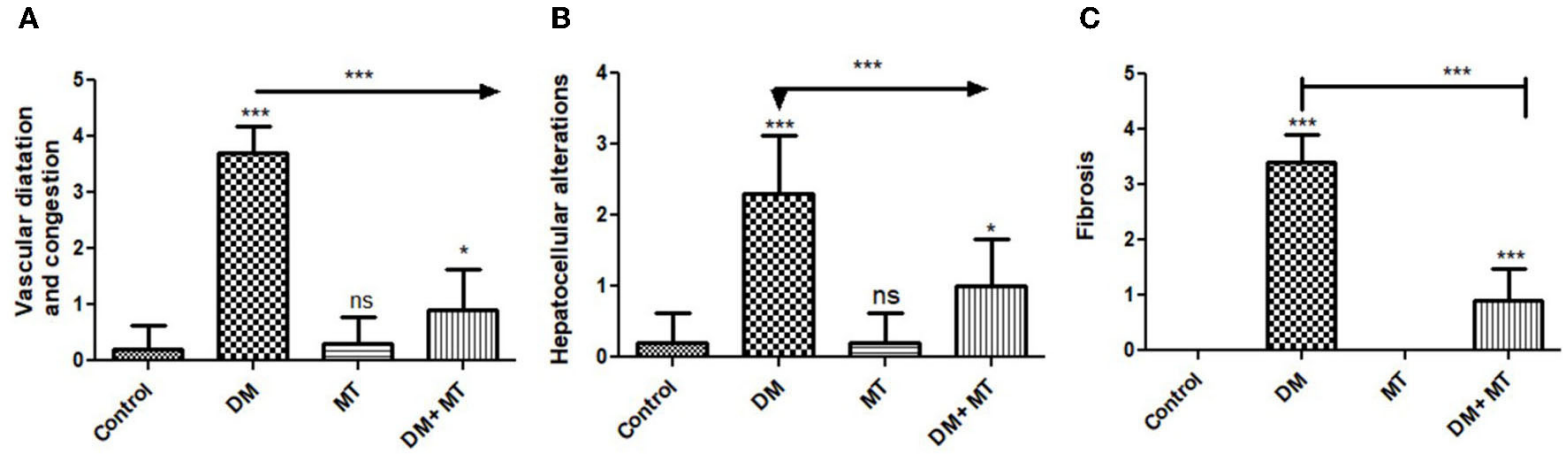
Histomorphometry graphs showing semiquantitative measurements of hepatic tissue sections among the experimental groups: **(A)** Hepatocellular alterations **(B)** vascular congestion **(C)** fibrosis. Data are expressed as means ± standard deviations. Significant differences vs the control group are marked by different asterisks through one-way ANOVA with Tukey's *post-hoc* test: *P ≤ 0.05, ***P ≤ 0.001.

Liver histomorphometric results showed significant (*P* < 0.05) cell damage in the liver tissue from the diabetic group, which was distinguished by hepatocellular degeneration+/-inflammatory cellular infiltration (Figure 8A), vasculatures, central veins, sinusoids, portal veins, congestion of hepatic arteries (Figure 8B), fibrosis, and the degree of its extension (Figure 8C) compared with other groups. In contrast, diabetic rats treated with MT displayed non-significant (*P* < 0.05) changes compared with the control groups and significant (*P* < 0.05) improvements in all assessed parameters compared with the DM untreated group.

#### 3.3.5. Immunohistochemical results

**Figure 9:**
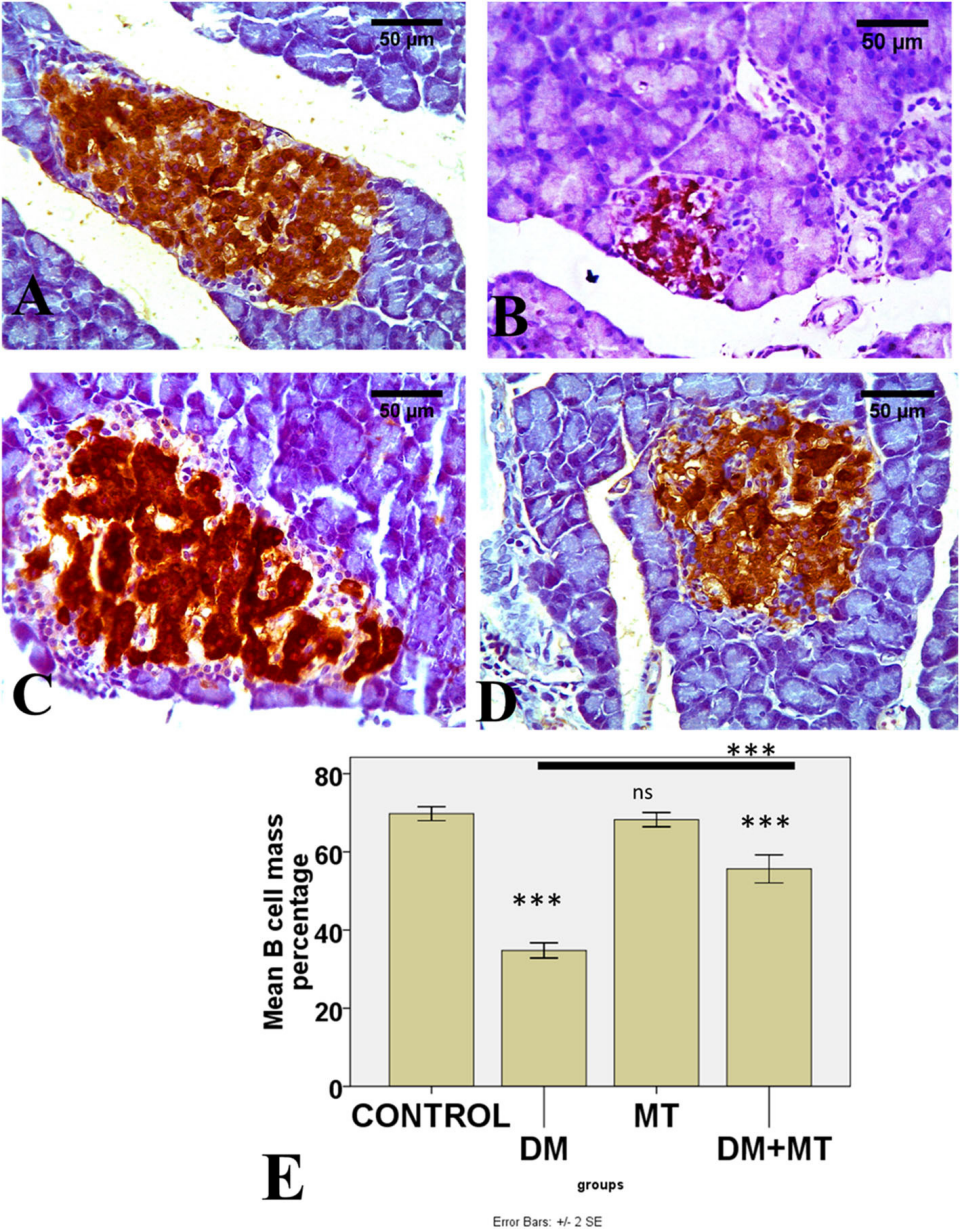
Photomicrographs of pancreatic sections of **(A)** Control and **(C)** MT group, both showing the expression of insulin in islet β-cells. **(B)** DM group showing decreased islet β-cells **(D)** DM+MT group showing increased islet β-cells. **(E)** Histogram representing the mean percentage area of insulin expression in all groups. Significant differences vs. the control group are marked by different asterisks through one-way ANOVA with Tukey's *post-hoc* test. ***P ≤ 0.001); ns, non-significant vs. control.

**Figure 10:**
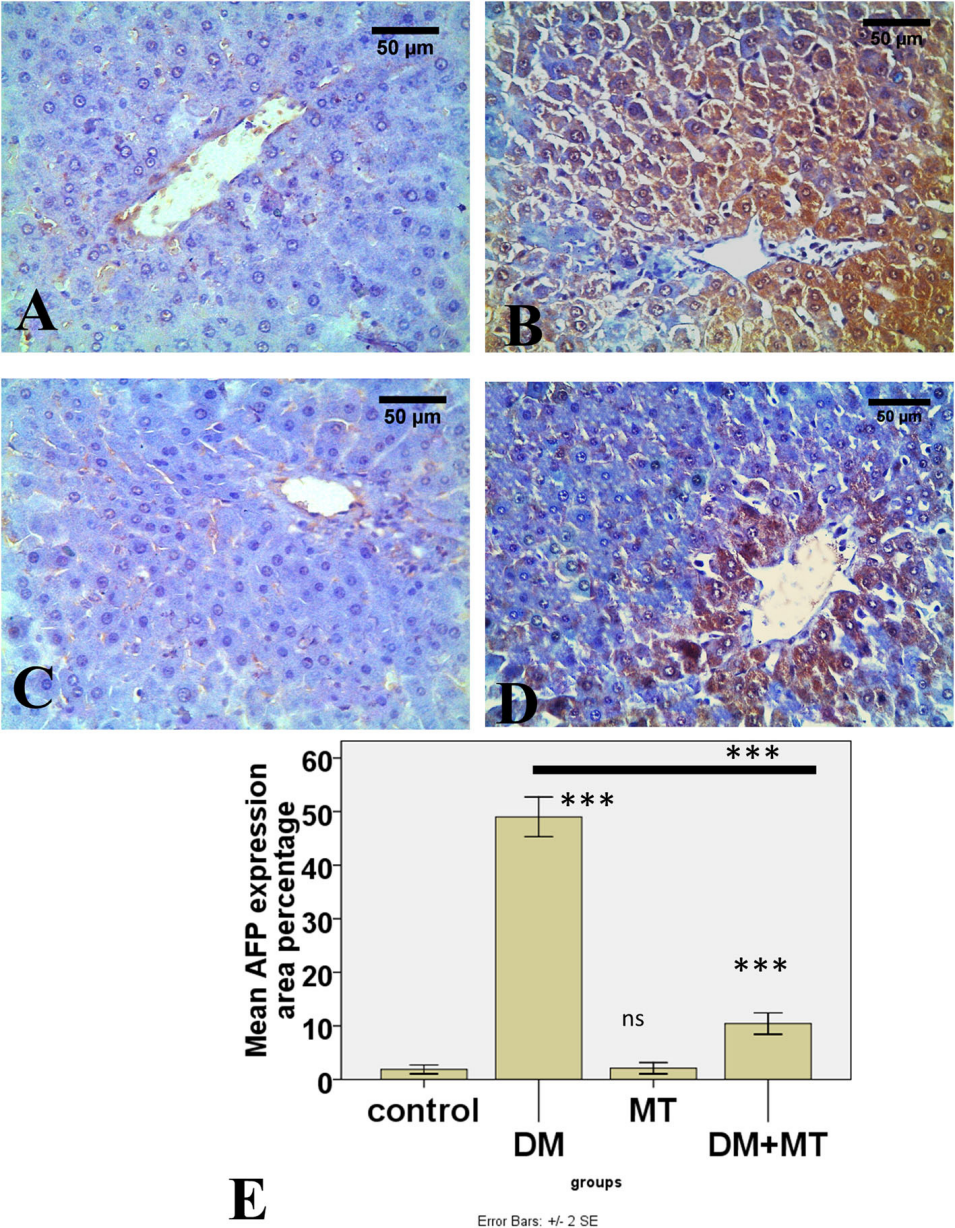
Photomicrographs of liver sections of **(A)** Control and **(C)** MT group, both showing minimal expression of AFP in hepatocytes **(B)** DM group. Showing the expression of AFP in most hepatocytes **(D)** DM+MT group showing the expression of AFP in few hepatocytes compared with the diabetic group. **(E)** Histogram representing the mean percentage area of AFP expression in all groups. Significant differences vs. the control group are marked by different asterisks through one-way ANOVA with Tukey's *post-hoc* test. ***P ≤ 0.001); ns, non-significant vs. control.

There was no significant difference between the negative control and MT groups in the β-cell area percentage of pancreatic islets. Contrarily, the β-cell area percentage was significantly (*P* < 0.05) decreased in the DM untreated group. The administration of MT in the DM+MT group increased the β-cell area percentage compared with the DM group. Furthermore, the improved expression of the β-cell area percentage was still significantly less than compared with the control group. Thus, melatonin had a protective effect on the pancreas of diabetic rats by repairing islet damage (Figure 9). There was no significant difference between the negative control and MT groups in the AFP area percentage in liver sections. When compared with the control and MT control groups, the DM group's AFP area % was significantly increased (*P* < 0.05). Compared with the diabetic group, the AFP area percentage of the DM+MT treated group was decreased, but it was still significantly (*P* < 0.05) increased when compared with the control group (Figure 10).

## 4. Discussion

Liver damage in patients with diabetes commonly occurs due to oxidative stress induced by hyperglycemia. Alpha-fetoprotein is a single-stranded glycoprotein primarily produced by the fetal liver and yolk sac. The level of AFP declines rapidly after birth and remains low throughout life. It is reactivated during liver regeneration and hepatocarcinogenesis (36). In our study, there was a significant decrease in body weight in the diabetic group compared with control. In accordance with our results (37), reported a loss of body weight in STZ-induced diabetic rats. In addition, a previous study (38) reported that body weight gain % was lower in the diabetic group compared with the control. Another previous study (39) reported lower body weight in diabetic rats compared with age-matched controls. The decrease in the body weight of diabetic rats may be due to an uncontrolled glucose level, muscle atrophy, and fat and protein breakdown (17). Different from our results, another study (40) reported an increase in body weight in diabetic animals. This could have been due to the combination of a high-fat diet and STZ in the induction of their diabetic model.

In the current study, the relative liver weight was increased in the diabetic group. This may have been caused by the increased influx of fatty acids into the liver due to hypoinsulinemia, resulting in the accumulation of triglycerides in the liver in addition to decreased lipoprotein secretion capacity of the liver due to apolipoprotein B synthesis deficiency (41). Additionally, our results revealed that the pancreatic weight and pancreas/body weight ratio were significantly decreased. Similar results were reported in previous studies (40), and they attributed the decreased pancreatic weight to the degeneration and atrophy of the islets, as observed later in our histopathological results.

In the current study, the fasting blood sugar level in the diabetic group significantly increased. In the diabetic group, the blood glucose level reached 362.0 ± 123.1 in the 4^th^ week of STZ injection. Similar results were reported by Ahmad and Ahmad (38). They reported a blood glucose level of 306.4 ± 2.65 in the 8^th^ week, which was the end of the experiment. In addition, Gomes et al. (37) observed increased blood glucose levels in the diabetic group in their study, which was attenuated upon treatment with the antioxidant *Trichilia catigua* (39).

In the present study, a significantly high HbA1c level was observed in the diabetic group, reflecting the diabetic status of rats over 1 month. Similarly, increased HbA1c levels have been observed in diabetic rats in several previous studies (38, 42, 43), and antioxidants such as thymoquinone (42) and *Acacia senegal* (43) could improve glycemic control. Long-term hyperglycemia can induce tissue damage, particularly in the liver, and liver impairment has been observed in individuals with diabetes whose blood sugar levels are uncontrolled (1).

In the current study, the serum concentration of insulin was significantly reduced in the diabetic group. However, no significant changes were recorded in these variables between the MT and control groups. Similarly, in previous studies, the insulin levels of diabetic rats were significantly decreased compared with the control group (38, 39). In our results, the ALT and AST levels were significantly increased in the DM compared with the control group. No significant change in ALT was observed between the MT and control groups, but a significant increase in AST was observed in the MT group compared with control. Consistent with our results, a previous study (44) observed systemic increases in ALT, AST, and ALP in diabetic animals, which were attenuated by the antioxidant naringin. Similar changes in diabetes have previously been shown in children and animal models (37, 45). A previous meta-analysis revealed a significant increase in the AST level in participants receiving treatment with MT compared with the control participants (46).

Elevated levels of AST and ALT reflect impaired glucose consumption. The changes in liver function markers such as AST and ALT are due to chronic stress in the liver of those with diabetes. Significantly, ALT and AST are two of the most critical enzymes engaged in the amino acid/glucose metabolism pathways and play a crucial role in amino acid metabolism and gluconeogenesis in several organs (47). Insulin deficiency leads to the breakdown of protein and enhances amino acid catabolism to provide substrates for gluconeogenesis, as evidenced by the decrease in total serum proteins (37). In addition, the increase in ALT and AST may be due to diabetes-induced hepatocyte degeneration, which causes these marker enzymes to release into the bloodstream (48). Liver function tests can reveal the extent of liver damage by describing the liver parenchymal cell integrity and the status of the biliary tract (1).

Furthermore, in the current study, the biochemical observations were parallel to the histopathological findings. The pancreas of the DM rats showed several histopathological degenerative changes, including distorted pancreatic lobules and necrosis in islets of Langerhans cells in the form of homogenous coagulated or vacuolated cytoplasm; the nucleus was pyknotic. Other locations were lysed and replaced by fatty tissues. In addition, capillary congestion was observed in between islet cells. A previous study (40) observed similar results in pancreatic islets but with the normal exocrine pancreas. These degenerative changes in pancreatic islets could be attributed to the cytotoxic action of STZ on pancreatic islet β-cells (42). The mechanism of toxicity of STZ depends on alkylation, followed by the fragmentation of DNA. This overstimulates poly (ADP-ribose) polymerase-1 (PARP-1), which in turn reduces cellular NAD+ and ATP and results in β-cell damage (49). Moreover, chronic hyperglycemia negatively affects pancreatic β-cells by inducing oxidative stress. β-cells possess the lowest intrinsic antioxidant activity, which makes them more sensitive to oxidative stress (39).

In our study, the exocrine parenchymal portion in the DM group showed atrophy, degeneration, and dissociation of some exocrine acini. The vascular system of the pancreatic tissue from this group generally revealed severe congestion with a very thick wall. In a similar study, diabetic rats showed pathological alterations in both the exocrine and endocrine pancreas. The acinar cells were swollen and vacuolated (50). The exocrine pancreas and β-cells could have been affected due to hyperglycemia-induced oxidative stress. In diabetes, ROS are formed through various pathways such as increased polyol, increased formation of advanced-glycation end-products (AGEs), and protein kinase C (PKC) activation (51).

In addition, we observed a significant decrease in the β-cell mass and the area of expression of insulin in the pancreas of the diabetic group by immunohistochemistry. This is in agreement with studies demonstrating that a single dose of 60 mg/kg STZ is capable of inducing β-cell damage with a subsequent decrease in insulin production (39, 52). In addition, a previous study (37) reported a decrease in the number of β-cells and the size of the islets in an STZ-induced diabetic rat model. The diabetogenic effects of STZ are due to the selective destruction of pancreatic islet β-cells (53). This could be explained by the cytotoxic action of STZ, which is selective to islet β-cells. The cytotoxicity is mediated by intracellular methylation reactions, DNA breaks, and ROS formation (42). Similar results were reported in a previous study of a high-fat diet + STZ diabetic model (40).

In our study, livers from DM untreated rats demonstrated central veins, and hepatic sinusoids between hepatic cords were dilated and congested. Furthermore, hepatocytes showed degeneration with diffuse mononuclear inflammatory cellular infiltration in some areas, and focal mononuclear cellular aggregation was also observed. The interlobular vein was distended and congested with blood. Furthermore, we observed that periportal fibrosis with the fibrous tract extended as a bridge connecting portal areas and surrounding the congested portal veins with inflammatory cellular infiltration. Similarly, in a previous study, livers from DM rats revealed disturbed hepatic architecture, pericentral sinusoidal dilatation, apoptosis, and lipid droplet accumulation in hepatocytes, in addition to signs of inflammation (44). Additionally, the degeneration of liver cells and the congestion of blood vessels with periportal fibrosis were reported in previous studies (48, 54, 55). Histopathological changes due to STZ administration observed in our study were similarly observed in previous studies (1, 56). Importantly, STZ and diabetes affect the liver possibly by inducing the release of ROS, which in turn leads to lipid peroxidation and membrane damage causing hepatocyte degeneration (49).

Liver damage in diabetic patients can be attributed to oxidative stress induced by hyperglycemia. Prolonged hyperglycemia increases ROS through the autoxidation of glucose. Consequently, disturbances in the metabolism of carbohydrates, proteins, and lipids occurred. These events, in turn, lead to the activation of inflammatory events cascades (3, 57, 58). Vascular dilation and congestion are triggered by ROS, which induces damage to sinusoidal endothelial cells. This in turn activates coagulation, causing sinusoidal obstruction, and ultimately, the dilation of interlobular vessels (49). In the current study, we observed a significant increase in AFP expression in the hepatocytes of diabetic rats compared with the control and MT groups by immunohistochemistry. This could have been due to hepatocyte regeneration resulting from diabetes-induced hepatocyte injury. Adult hepatocytes re-express AFP mainly when they act as functional stem cells and have the capacity for regeneration after exposure to injury. When hepatocytes regenerate, the AFP levels increase (5). Endoplasmic reticulum stress and hepatic cholestasis in diabetes lead to hepatocellular injury. Lipid accumulation leads to hepatic steatosis (59). There is a correlation between having high AFP levels and insulin resistance, which may be caused by hepatic steatosis. Hepatocytes play a significant role in the regulation of glucose homeostasis, and depending on the demands of the body, they either create or store glucose. Insulin resistance affects hepatic glucose homeostatic pathways, which in turn causes free fatty acids to release from adipose tissues and leads to an increase in the creation of very low-density lipoprotein by the liver (60).

Melatonin (N-acetyl-5-methoxytryptamine) is synthesized in the pineal gland. It acts as a neuroendocrine transducer and regulates the day/night cycle. It regulates the physiological synchronization of glucose metabolism and stimulates insulin secretion (GSIS) as well. It mediates various signaling pathways in pancreatic islets through two membrane receptors (MT1 and MT2). Being lipophilic in nature, it diffuses easily through biomembranes and the nucleus. Previous studies have reported that MT is a powerful antioxidant in biological systems and acts as an immune regulator (61).

In our study, MT restored the body weight of diabetic rats. This occurred due to the administration of MT, which increased the glucose decay constant and improved insulin sensitivity. Melatonin stopped the aberrant glycosylation of proteins and the consequent weight loss. The lipolysis of adipose tissues may have been inhibited by the action of insulin, which may have restored the previous protein content levels in the cells and tissues. Along with its ability to scavenge free radicals, MT also prevents the aberrant glycosylation of proteins that would otherwise be caused by oxidative stress (62). Similar to our study, in another study, MT restored body weight, which was significantly decreased in diabetic rats compared with the control (61).

In our study, at the end of the fourth week, MT significantly reduced the cumulative blood sugar level in the DM+MT treated group when compared with the diabetic group. However, it was still significantly increased compared with control. In addition, there was a significant increase in insulin in the DM+MT compared with the DM group, which explains the decrease in the blood glucose level. This could be attributed to the fact that MT can promote insulin production by acting through the cAMP pathway (17). In contrast, previous research has shown that MT can reduce the amount of insulin that the body produces by acting through a pathway regulated by the MT receptors MT1 and MT2. It decreases insulin secretion by inhibiting cAMP and cGMP pathways. However, it activates the phospholipase C/IP3 pathway, which mobilizes Ca2+ from organelles with a consequent increase in insulin secretion (63). Similar to our results, the treatment of diabetic rats with MT significantly reduced their blood glucose, HbA-1c, and insulin levels compared with untreated diabetic rats; however, the levels were still higher than those observed in the control group in previous studies (7, 17, 61).

In the present study, the concomitant administration of MT to DM rats in Group IV ameliorated the previously mentioned pathological degenerative changes observed in the DM group. Melatonin restored the normal architecture of pancreatic lobules. We observed normal cellular density and morphology of islets of Langerhans and the exocrine parenchyma. Similar results were reported elsewhere (7). This could be due to the ability of MT to induce β-cell proliferation, in addition to its antioxidant effect. It does this by scavenging ROS and reactive nitrogen species (RNS). In addition to this, MT suppresses the activity of certain prooxidant enzymes, such as NADPH oxidase, while simultaneously activating the expression pattern of antioxidant enzymes, such as glucose-6-phosphate dehydrogenase (G6PDH) and superoxide dismutase (SOD). Being an electron-rich molecule, it can also neutralize free radicals, which causes the reduced level of glutathione (GSH) to increase (64).

In conclusion, our study revealed that MT can restore the normal liver histological structure and the decreased liver enzymes ALT and AST. Consistent with our results, in a previous study, MT administration showed recovery in histopathological alteration and liver enzymes (17, 65). This might be attributed to the importance of the insulin–MT relationship (17). We also observed decreased AFP expression with MT administration in diabetic rats due to attenuated liver cell injury. Similarly, MT decreased the AFP level in a hepatocarcinogenesis model (4).

Taken together, the present study concluded that MT treatment regulates the blood glucose level, controls liver enzymes ALT and AST, and attenuates diabetes-induced hepatic injury by restoring pancreatic β-cells and their antioxidant effect, which decreases hepatocyte injury and downregulates AFP expression. Our study establishes the promising role of MT in the treatment of diabetes.

## Data availability statement

The original contributions presented in the study are included in the article/supplementary material, further inquiries can be directed to the corresponding authors.

## Ethics statement

This study and all experimental procedures were performed according to the principles of the Ethics Committee of Taif University, Taif, Saudi Arabia (Approval No. HAO-02-T-105) which are in line with the Declaration of Helsinki.

## Author contributions

FA, SM, DA, and EE contributed to the experimental design, sampling, histopathological and morphometrical assessments, wrote the manuscript draft, revised the final manuscript, and contributed in response to the reviewers. KAls, MAlb, AH, FA, SM, and AAlbr were involved in the conception of the idea, methodological design, and manuscript preparation for publication and revision. AT, OA-A, MAlm, AAlm, AAlba, KAlz, and MAlba were involved in the methodological design, data analysis performance and interpretation, and manuscript preparation for publication. All authors have read and approved the final manuscript.
